# Delayed Discharge for Non-Clinical Reasons in Hip Procedures: Differential Characteristics and Opportunity Cost

**DOI:** 10.3390/ijerph18179407

**Published:** 2021-09-06

**Authors:** Amada Pellico-López, Ana Fernández-Feito, David Cantarero, Manuel Herrero-Montes, Joaquín Cayón-De Las Cuevas, Paula Parás-Bravo, María Paz-Zulueta

**Affiliations:** 1 Cantabria Health Service, Avda. Derechos de la Infancia 31, 39340 Suances, Spain; amada.pellico@scsalud.es; 2 Department of Medicine, Faculty of Medicine and Health Sciences, University of Oviedo, Avda. Julián Clavería s/n, 33006 Oviedo, Spain; 3 ISPA, Nursing Research Group, Health Research Institute of Asturias, Avda. del Hospital Universitario s/n, 33011 Oviedo, Spain; 4 Department of Economics, University of Cantabria, Avda. de los Castros s/n, 39005 Santander, Spain; david.cantarero@unican.es; 5 IDIVAL, Research Group of Health Economics and Health Services Management, Research Institute Marqués de Valdecilla, C/ Cardenal Herrera Oria s/n, 39011 Santander, Spain; 6 Faculty of Nursing, University of Cantabria, Avda. Valdecilla s/n, 39008 Santander, Spain; manuel.herrero@unican.es (M.H.-M.); paula.paras@unican.es (P.P.-B.); maria.paz@unican.es (M.P.-Z.); 7 IDIVAL, Grupo de Investigación en Enfermería, C/ Cardenal Herrera Oria s/n, 39011 Santander, Spain; 8 Faculty of Law, University of Cantabria, Avda. de los Castros s/n, 39005 Santander, Spain; joaquin.cayon@unican.es; 9 IDIVAL, GI Derecho Sanitario y Bioética, GRIDES, C/ Cardenal Herrera Oria s/n, 39011 Santander, Spain

**Keywords:** hip fractures, hip injuries, hospital costs, length of stay, patient discharge, economic burden

## Abstract

Delayed discharge for non-clinical reasons shares common characteristics with hip procedures. We sought to quantify the length of stay and related costs of hip procedures and compare these with other cases of delayed discharge. A cross-sectional study was conducted at a public hospital in Spain (2007–2015) including 306 patients with 6945 days of total stay and 2178 days of prolonged stay. The mean appropriate stay was 15.58 days, and the mean prolonged stay was 7.12 days. The cost of a prolonged stay was €641,002.09. The opportunity cost according to the value of the hospital complexity unit was €922,997.82. The mean diagnostic-related groups’ weight was 3.40. Up to 85.29% of patients resided in an urban area near the hospital (*p* = 0.001), and 83.33% were referred to a long-stay facility for functional recovery (*p* = 0.001). The proportion of patients with hip procedures and delayed discharge was lower than previous reports; however, their length of stay was longer. The cost of prolonged stay could account for 21.17% of the total. Compared with the remaining cases of delayed discharge, the appropriate stay was shorter in hip procedures, with a profile of older women living in an urban area close to the hospital and referred to a long-stay center for functional recovery.

## 1. Introduction

The phenomenon of delayed discharge for non-clinical reasons is defined as “a period of continued stay after a patient is deemed medically fit to leave hospital but is unable to do so for non-medical reasons” [1]. The prevalence of this problem varies significantly across studies, depending on the context, ranging from 1.6% to 91.3%, with a mean of 22.8%, and affecting all types of health systems, including public health systems and those that are mainly funded by private insurance [2]. Compared to continuous monitoring in other regions of Europe such as the United Kingdom, where reports suggest up to 8.5% number of stays affected by delayed discharge [3], studies on this problem are scarce in Spain, with a recent study by our own research team finding 0.93% of cases out of the total number of discharges in a hospital of high complexity [4]. 

Advanced age is a patient characteristic that is closely related to a greater probability of delayed discharge for non-clinical reasons [5,6,7,8,9,10,11,12,13,14,15]. Beyond the likelihood of delayed discharge, the relationship between age and length of stay is unclear in the literature consulted. Some studies have clearly found longer delays in older patients, but the relationship between age and length of stay is unclear [5,16]. In contrast, other studies relate younger age of cases with delayed discharge to longer length of stay [17,18]. Therefore, it is relevant to conduct studies in regions of northern Spain where an increase in the regional aging rate was observed during the study period, concretely increasing from 18.57% in 2007 to 20.27% in 2015 [19]. 

Overall, this problem is associated with pathologies of greater clinical complexity [13,15,20], comorbidity [13], loss of functional capacity and dependency [12,21], or with added social risk such as those with cognitive impairment [9,12,13,14,21]. No conclusive results have been found in terms of the role of gender [5,15,18]. From the point of view of the care context, delayed discharge appears to be more likely in urgent admissions [14], when surgery is required [5,12], in hospitals of high complexity [20], when there is a need for functional recovery and post-hospitalization rehabilitation [12,21], or if the patient is discharged to a nursing home [7,11,12]. At the family level, the lack of a primary caregiver is influential, as well as the caregiver’s inability to assume care after discharge [21,22,23]. Additionally, patients who live on their own or have a weak social support network have a higher risk [6,9]. 

All the above-mentioned factors are of particular significance in pathologies associated with aging and frailty, such as hip fracture. This is a pathology clearly associated with loss of quality of life among older people, increased mortality, and greater healthcare costs. Recent studies estimate an annual incidence in Spain of 104 cases per 100,000 inhabitants, with a cost of 1591 million euros and a loss of 7218 quality-adjusted years of life [24]. The impact of surgical procedures on the hip in terms of incidence and costs in our health system is important. That is why this study has sought to understand in detail the characteristics of delayed discharge for non-clinical reasons in patients who require these procedures. This will allow us to outline solutions for more efficient use of acute hospitalization in these cases.

The Spanish healthcare system is characterized by its universal coverage and tax-based funding, as well as the provision of long-term care after acute hospitalization. During the economic crisis in Spain, this period of study coincided with the implementation of the system of care for dependent persons, which supports care at a family and community level, is associated with a co-payment based on the user’s income level and has demonstrated an impact in reducing the average length of stay in the event of hospital admission [25]. The analytical accounting systems of Spanish public hospitals during the study period were based on the data collected in the minimum basic data set at hospital discharge (MBDSHD) for each case and its classification according to diagnostic-related groups (DRGs), enabling an evaluation of the efficiency of hospital stay as a public resource.

We hypothesized that delayed discharge for non-clinical reasons in patients with hip-procedures (HPs) is a relevant problem with a high impact on the efficient use of acute hospitalization wards. Hip pathology remains as prevalent in the current pandemic situation as it was in the study period, aggravated today by the fact that the use of hospital beds is of great priority and inappropriate hospitalization adds considerable iatrogenic exposure in patients who are particularly vulnerable. The aim of the present study was to quantify the length of stay and related costs of HP with delayed discharge for non-clinical reasons. Likewise, this group was compared with the rest of the cases of delayed discharge in terms of differences in length of stay, costs, patient characteristics, and factors specific to the context of care.

## 2. Materials and Methods

### 2.1. Study Location and Population

A cross-sectional study, covering the period from 1 January 2007 to 31 December 2015. During this period the implementation of the system of care for dependent persons in Spain coincided with a major economic recession, during which the efficient use of each level of the care network was of particular concern.

The study setting was the Marqués de Valdecilla University Hospital, in Santander (Cantabria, northern Spain). This is a publicly owned hospital, with teaching accreditation, which had 903 inpatient beds at the end of the study period [26] and directly served a population of 319,751 users and represented a national reference for certain highly qualified healthcare and technological services [27]. The hospital under study systematically collected both the date of medical discharge and the date of actual discharge, both of which are essential sources for determining the length of stay.

The study population was the total number of cases with delayed discharge for non-clinical reasons during the period of study. The study included all those patients identified as ready for medical discharge by the hospital admission department, but whose actual discharge was delayed by more than 24 h. Patients discharged to other hospitals or under the care of the hospital’s own home hospitalization service were excluded. 

The sample size calculation was based on a recent review of the proportion of delayed discharge for non-clinical reasons in different countries, which shows very wide variability, from 1.6% to 91.3% [2]. This is in line with preliminary data from the same research group in a pilot study in the same hospital, which found 1567 cases during the years 2010–2014. With this expected proportion interval, a calculation was made of how many patients would have to be recruited annually considering the number of hospital discharges in the study period according to data from the regional health service [26]. A confidence level of 95% and a precision of sampling error of 1% were estimated. As an example, in 2015, for a population of 36,471 discharges, it was estimated that it would be necessary to gather between 60 and 282 cases to ensure the statistical power of the study. 

The study data were collected based on the information provided by the hospital’s Admission and Analytical Accounting Services. Of the total number of cases of delayed discharge for non-clinical reasons, patients with a hip procedure were isolated for comparison with the rest. The DRGs included in this group were those involving surgical procedures on the hip joint (Appendix A). The coding of the DRGs was version 25.0, which was valid at the end of the study period [28]. Among the variables compared, a differentiation was made between those related to the length of stay and costs, those related to the patient, and those related to the care process. 

### 2.2. Variables

Length of stay: the duration in days of appropriate stay (between the date of admission and medical discharge), prolonged stay (between the date of medical discharge and actual discharge), and total hospital stay (sum of the above) was calculated. The date of medical discharge is established from the date at which the medical team in charge of the patient issues the discharge report, a document certifying that the process for which the patient was admitted has been resolved. The actual discharge date is the day on which the patient actually leaves the hospital.

The difference between the total stay of the cases found and what would have corresponded for the same DRG and year of discharge according to the hospital’s own data (corrected prolonged stay) was also considered. This calculation allows the correction of a possible bias due to a covert delay due to inaccurate records.

Costs: the cost of appropriate stay was calculated from the cost of each day of stay according to the DRG (Table 1). The cost of prolonged stay depended on the cost of the stay in the hospitalization unit and the opportunity cost of the value according to the hospital complexity unit (HCU). A hospital complexity unit is a unit used in Spain to determine the financing of hospitals in each region. It measures the cost of hospitalization activity, weighting in relative terms the complexity of the pathology in admitted patients. It is calculated by multiplying the number of discharges by the average hospital weight according to DRG.

Patient characteristics: the patient variables that were gathered included age, sex, and relative weight of the DRG, to determine the complexity of the process. 

Process variables: we recorded the type of admission (urgent or programed), urban or rural place of residence (urban corresponding to residents in the same region than hospital and with more than 50,000 habitants and with a density of more than 1500 residents per km^2^, rural to the rest of the regions), year of medical discharge (2007–2015), and discharge destination (long-term care center, home, death during the period of prolonged stay or nursing home for dependent people). 

### 2.3. Data Analysis

All data were analyzed using R 3.6.0 for Windows (R Foundation, Free Software Foundation, Boston, MA, USA). The economic impact of each length of stay was quantified by multiplying the number of days by the corresponding cost (DRG or hospitalization unit). The calculation of the opportunity cost was obtained by multiplying the number of patients who could not be attended in that period (estimated according to average stay) by their DRG weight and the cost corresponding to that DRG weight according to the hospital complexity unit. The calculation was made according to two models. Model 1 of the calculation was based on the real data and model 2 on the prolonged stay, assuming that this was the difference between the total stay and the appropriate stay that would have corresponded for the same DRG and year of discharge according to the hospital’s own data (corrected prolonged stay).

In the descriptive analysis, proportions with their corresponding 95% confidence intervals (95% CI) were estimated for the discrete variables. For the continuous variables, means and standard deviation (SD) were estimated. To compare the differential characteristics of the groups of patients with hip procedures with the total cases of delayed discharge for non-clinical reasons, continuous quantitative variables were compared using Student’s *t*-test and Pearson’s chi-squared test (χ^2^) for categorical variables. Adjustment for multiple comparisons was made by applying the Bonferroni correction, considering a *p*-value less than or equal to 0.0015 as significant.

## 3. Results

The descriptive data of the cohort are published elsewhere [4]. A total of 306 patients with HP and delayed discharge for non-clinical reasons were identified during the study period. 

These cases accumulated a total of 6945 days of total stay, of which 2178 days corresponded to a prolonged stay. The mean total hospital stay was 22.69 days [SD 29.65]. The mean length of appropriate stay was 15.58 days, and the mean length of prolonged stay was 7.12 days. About 27.8% (95% CI 22.83–33.16) of the cases had a prolonged stay of only one day. Comparing the total stay of these cases with what would have corresponded for the same DRG and year of discharge, the mean of this difference was 9.58 [SD 27.25] additional days of stay. 

**Figure 1:**
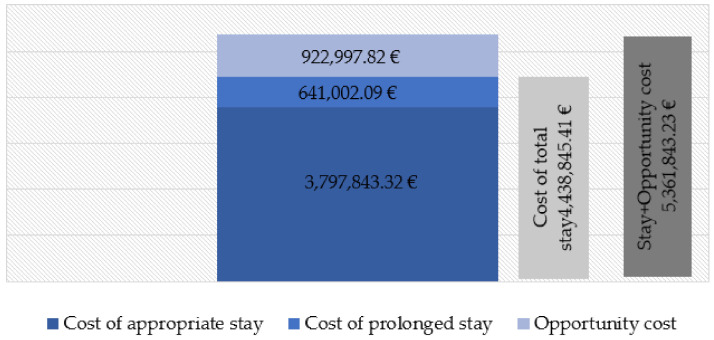
Costs of delayed discharge for non-clinical reasons in hip procedures. Model 1: according to actual hospital stays. Cantabria (northern Spain), 2007–2015.

**Figure 2:**
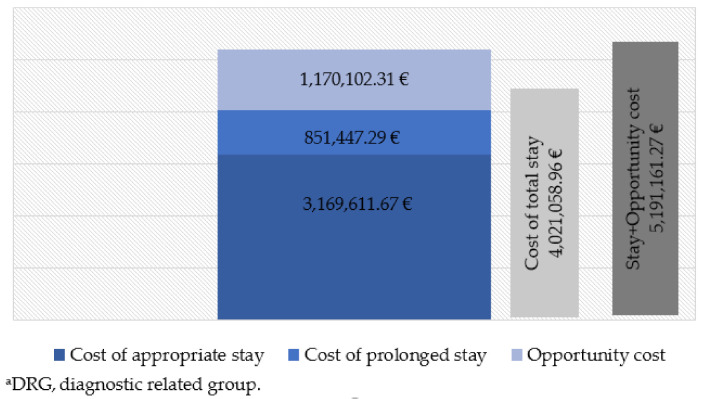
Costs of delayed discharge for non-clinical reasons in hip procedures. Model 2: on the basis of hospital stays that would correspond to the same DRG^a^ and year. Cantabria (northern Spain), 2007–2015.

Figure 1 and Figure 2 show the costs corresponding to the hospital stay of the cases during the study period. The cost of the appropriate hospital stay of the cases equaled a total of €3,797,843.32 according to the DRG process. The cost of prolonged stay amounted to 641,002.09 (14.44% of the total); however, considering the prolonged stay that would have corresponded to the same DRG and year of discharge, it amounted to 851,447.29 (21.17% of the total). The total cost resulting from the sum of both periods is 9.41% higher if the actual hospital stay is considered. The estimated opportunity cost according to the value of the UCH of the prolonged stay period was €922,997.82, although, once again, considering the prolonged stay that would have corresponded for the same DRG and year of discharge, it amounted to €1,170,102.31. Finally, the total cost was 3.18% higher, considering actual hospital stay.

Table 2 shows the results in terms of length in days of each hospital stay and costs of the cases of delayed discharge for non-clinical reasons with HP, compared with the remaining cases that appeared during the study period. Patients admitted for HP had shorter appropriate and total hospital stay than the rest (*p* < 0.001); however, there were no differences in the hospital stay or in the costs associated with each period according to real data, nor in the opportunity cost. 

Table 3 shows the results describing the characteristics of the patients and their context of care in the cases of delayed discharge for non-clinical reasons with HP compared with the rest of the cases that appeared during the study period. 

Regarding patient characteristics, 75.82% of the cases of delayed discharge with HP were women, a proportion with statistically significant differences over the rest of the cases (*p* < 0.001). The mean age was 82.73 years, and significantly higher (*p* < 0.001) than the remaining patients, who had a mean age of 76.78 years. Up to 13.2% (95% CI 12.08–20.61) of the cases with HP were 75 years old or younger. The mean complexity of the cases according to the DRG weight value was 3.401, and there were no differences with the remaining cases with delayed discharge. 

Regarding the process of care, 85.29% of patients with HP resided in the urban area near the hospital, a significantly higher proportion, compared to the remaining patients (*p* < 0.001). Emergency admissions were 90.85%, with no differences, compared to other cases. Regarding discharge destination, 83.33% of patients with HP were significantly more likely (*p* = 0.001) to be referred to a subsidized long-stay center, and only four patients with HP died during the delayed discharge period, a proportion significantly lower than the remaining patients. No differences were observed in the progression of cases over the years of the study period, although 2008 was the year with the highest number of cases and 2015 was the year with the lowest.

## 4. Discussion

According to our hypothesis, delayed discharge for non-clinical reasons in patients with hip procedures is a relevant problem, representing one of the most prevalent diagnoses in cases of delayed discharge for non-clinical reasons during the 2007–2015 study period and with a high impact on the efficient use of the acute hospitalization ward. Hip-procedures and the musculoskeletal category, in general, were the most relevant in delayed discharge, together with stroke or other nervous system diagnoses [4].

The cost of prolonged stay represented close to 21.17% of the total cost of stay. Additionally, it is important to consider the opportunity cost, which also has an economic impact and is a cause of disability and dependence.

The 306 patients with a hip procedure who suffered delayed discharge due to non-medical reasons corresponded to 4.18% of the 7317 discharges with the same DRGs related to hip procedures during that period. This prevalence is significantly lower than that found in other studies measuring the problem in HP patients [23]. A record of the date of discharge based on clinical criteria provides an accurate measure of the stay in cases of delayed discharge [29]. Despite the concern in Spain about the average stay as an indicator of hospital efficiency, there are no objective criteria in our country on when to consider that the patient is clinically fit for discharge, as opposed to what happens in other countries [30]. The fact that about a third of cases have a prolonged stay of only one day suggests that medical discharge may depend on our cases having found a post-hospitalization solution to the loss of functional capacity and not so much on clinical criteria.

In our study population, 15.58 days corresponded with the mean of the appropriate hospital stay. Regarding appropriate hospital stay, according to data from the hospital analytical accounting systems, the mean hospital stay for all cases discharged from the hospital in the same period with the same DRGs was 12.43 days, slightly less than that found in our study. Our results indicate that both the total and appropriate hospital stay were longer in terms of the number of days, compared to other studies, such as the mean length of stay of 10.9 days reported in a national multicenter study of hip fracture patients [24] or the mean total length of stay of 13.1 days obtained by Landeiro et al. in their study [23]. Under the assumption of an unrecorded covert delayed discharge, this was compared with cases with the same DRG and year of discharge without delayed discharge, in which case, the prolonged stay would be 9.58 days. Therefore, the data on appropriate hospital stay would be more similar to those of other authors [23,24], reinforcing the theory of a covert delay due to inaccurate records.

In our study, prolonged stay (7.12 days) accounted for more than one-third of the total stay, compared to 11.5% in the study by Landeiro et al. [23], showing a late response to the problem that adds further inefficiency to the system. 

When evaluating the costs of this problem, and considering the data, the cost of the total hospital stay was higher (€4,438,845.41) than if one were to consider the prolonged stay that would have corresponded for the same DRG and year of discharge (€4,021,058.96). Therefore, when correcting for the bias due to the effect of discharge when there is already a solution, a lower estimate is made of the total cost, probably because the period of prolonged stay is longer and therefore cheaper. This effect is because during the days of prolonged stay the patients are occupying an acute hospitalization bed that they no longer need, and therefore, they should not be charged for the costs of an appropriate stay such as surgery fees, radiology, or laboratory tests, etc. In those days of prolonged stay, “hotel costs” are attributed to the patient, corresponding to the cost per unit of hospitalization. This same criterion was used in a study published in 2013 by Holmas et al. [20]. 

Holmas et al. (2013) [20] consider that it is important to account for the patients that the hospital stops treating due to this prolonged stay. In this opportunity cost, the opposite is true regarding the costs of the prolonged stay. In this case, the actual prolonged stay of the cases in the study period amounts to €922,997.82. However, to correct the bias due to the effect of discharge when there is already a solution, using the model based on the difference between the hospital stay of the cases and the hospital stay that would have corresponded to each DRG and year, an opportunity cost of more than double (€1,170,102.31) was estimated. To quantify this opportunity cost, we used the cost per HCU. The HCU is a measure used to determine hospital financing. The greater the capacity to resolve complex cases in the hospital under study is, the higher is the cost of the HCU, and the higher is the opportunity cost. 

In addition to the direct costs, we must consider indirect costs (disability, dependence on third parties, or sick leave) for patients suffering from the surgical waiting list for trauma procedures. Specifically, according to data from the regional health service, in December 2015, there were 3186 people awaiting surgery for traumatology, waiting for an average of 113 days. Overall, 18.4% had been waiting for over six months [31]. Although delayed discharge affects any type of health system regardless of its public or private financing, Spain is a country with tax-based financing and universal coverage with data on waiting lists for major surgery that make its health system to be ranked below the European average [32].

Comparing the hospital stay and costs of patients admitted for HP with the remaining patients, the former have a shorter appropriate and total length of stay (*p* <0.001), with no differences in prolonged stay or in the costs associated. Despite the existence of a concealed delay, the fact that the appropriate hospital stay, and therefore the total hospital stay, are shorter than other procedures may be due to the fact that hip surgery is highly standardized [33] and is more easily detected when the therapeutic possibilities have been exhausted and the patient can be referred to another level of care, as opposed to what happens in medical procedures. 

Regarding patient characteristics, 75.82% of patients with HP were women, a proportion significantly higher than other cases. This relationship with the female sex is already known and coincides with other studies on HP [23,24,34]. 

The mean age of the cases with a hip procedure was 82.73 years, significantly higher than the rest of the cases. Although this is an advanced mean age, it is still lower than other studies referring to HP, which report mean ages above 85 years [23,24], while the very wide range and the fact that 13.2% of cases were younger than 75 years are remarkable findings. 

The mean complexity of our cases according to the DRG weight value was 3.401, with no differences in relation to the remaining patients. This value reflects the consumption of resources used by the hospital to care for its patients, based on the mean annual cost of hospitalization in acute units (weight = 1) [35]. According to hospital analytical accounting systems data, the mean annual complexity ranged from 1863 to 1949. The increase in the level of complexity is due to the surgical process, additional procedures, and secondary diagnoses quantified in the DRG that increase complexity, which, according to previous studies, is related to a longer hospital stay [4,15,20].

Regarding the process of care, most cases were admitted urgently, with no differences compared to the rest of the cases. Therefore, 90.85% of the cases were acute and unexpected, compared to programmed interventions for hip osteoarthrosis. Other studies consulted focus on the fracture and not on the total number of hip procedures, as in our case [23,24]. 

Of the cases with HP, 85.29% resided in the urban area near the hospital, a significantly higher proportion compared to the rest. Other authors have found a relationship between delayed discharge and residing in the same area as the hospital, due to the use of the hospital as a temporary resource for stay in the absence of other social and health services [20]. Regarding discharge destination, 83.33% of patients were significantly more likely to be referred to a long-stay center offering rehabilitation after the discharge of patients with loss of functional capacity. According to the studies consulted, the availability of long-stay centers in the patients’ area of residence makes it more likely that they will be referred to a resource of this type after discharge for hip fracture [15]. The proportion is much higher than that reflected in a nationwide study that found 23.8% of patients referred to geriatric rehabilitation units [24]. The situation may reflect a delay caused by a “bottleneck” in the availability of a place at that long-stay center. 

Only 1.31% of patients with HP died during the period of prolonged stay, a significantly lower proportion than the rest of the cases. National studies estimate mortality due to hip fracture at 7.1% in the first month [24]. Given the appropriate hospital stay of the cases, possibly, deaths occur early without the patient being considered a case of delayed discharge.

No differences were observed in the progression of cases over time, although 2008 was the year with the highest number of cases and 2015 had the lowest. This finding coincides with studies that demonstrate the effect of the implementation in Spain of the system of care for dependent persons on hospital stay [25], the greater availability of caregivers due to unemployment resulting from the economic recession in the country [21,23], or the implementation of organizational measures in this period [36]. 

Early discharge planning is proposed as the best solution that has shown positive results in the reduction of prolonged stay and subsequent readmissions with high satisfaction of professionals, family, and patients [37]. The professionals can identify patients at risk for early referral to the social worker and search for a discharge resource from the moment of admission, minimizing the hospital stay [38].

Long-term facilities for functional recovery have been considered adequate to favor the transition between acute hospitalization and return to home or to the nursing home [39]. This resource has been shown to reduce hospital stay without increasing readmissions, constituting a more efficient resource than traditional hospitalization [40].

Regarding the study limitations, the variables are based on data collected using the MBDSHD. Using such records, in addition to guaranteeing systematically collected data, enabled the handling of a large amount of data from a broad period. However, regarding the process of patient care, other variables that have been shown to be related to the problem, such as cognitive impairment, lack of social or family support, previous residence alone, or increased level of dependence for self-care, are collected in the patient’s clinical history [23,24]. However, these data are not objectively reflected in the MBDSHD, and the loss of this information requires a review of the information recorded by the professionals in the clinical history. Similarly, there are no data in this information system that enable us to objectively quantify the indirect costs of delayed discharge, such as the loss of functional capacity of the patient waiting to undergo surgery due to the lack of a bed, blocked by delayed discharge. Our study covers all hip pathology under the term hip procedures, which encompasses any surgical procedure affecting the hip joint, including different etiologies such as fractures or arthrosis arthroplasty of the joint. This makes it difficult to compare with studies focused only on fractures [23,24]. This report is based on the results of a nine-year period in a single hospital. In other hospitals in the region, there were different criteria for considering a case as a delayed discharge for non-clinical reasons, and the hospital under study was the only one that systematically collected the date of medical discharge and the date of actual discharge, both of which are essential information for calculating the length of stay. This difference in criteria probably also exists between hospitals at the national level, making the comparison between regions difficult.

## 5. Conclusions

The proportion of patients with HP and delayed discharge was lower compared to other studies. The appropriate hospital stay was longer in our cases. The prolonged stay had a cost of 14.44% of the total; however, considering the cost that would have corresponded to the same DRG and year of discharge, it could amount to 21.17% of the total. 

Compared to the rest of the cases of delayed discharge, in patients with HP, the appropriate hospital stay was shorter, characterized by a profile of older women living in an urban area close to the hospital and referred to a long-stay center for functional recovery. 

## Figures and Tables

**Table 1:** Calculation formulas for the costs of each period of stay. Cantabria (northern Spain), 2007–2015.

**Cost of appropriate stay** = Days of appropriate stay × cost stay per DRG ^a^
**Cost of prolonged stay** = Days of prolonged stay × cost stay in hospitalization unit
**Opportunity cost** = ((Prolonged stay/Average length of stay) × Average DRG weight ^a^) × Cost according to HCU ^b^

^a^ DRG, diagnostic related group; ^b^ HCU, hospital complexity unit.

**Table 2:** Patients with hip procedures compared with patients without hip procedures (length of stay and costs). Cantabria (northern Spain), 2007–2015.

	Hip Procedures (*n* = 306)	SD ^a^	Non-Hip Procedures (*n* = 2709)	SD ^a^	*p*-Value
Total stay (days)	22.69	[29.65]	29.18	[30.08]	<0.001
Appropriate stay (days)	15.58	[21.13]	21.82	[23.31]	<0.001
Prolonged stay (days)	7.12	[13.78]	7.36	[16.11]	0.800
Cost of appropriate hospital stay (euros)	12,411.25	[13,899.74]	15,237.43	[24,609.78]	0.049
Cost of prolonged stay (euros)	2094.78	[4250.07]	2128.64	[4482.92]	0.900
Total cost of hospital stay (euros)	14,506.03	[16,029.90]	17,366.07	[25,577.87]	0.056
Opportunity cost (euros)	3016.33	[4265.95]	3432.74	[6832.01]	0.297

^a^ SD, standard deviation.

**Table 3:** Patients with hip procedures and compared with patients without hip procedures (patient characteristics and context of care). Cantabria (northern Spain), 2007–2015.

		Hip Procedures (*n* = 306)	SD ^a^, 95% CI ^b^	Non-Hip Procedures (*n* = 2709)	SD ^a^, 95% CI ^b^	*p*-Value
Sex	Male	74 (24.18%)	(19.49–29.39)	1370 (50.57%)	(4867–5247)	<0.001
	Female	232 (75.82%)	(70.62–80.51)	1339 (49.43%)	(47.53–51.33)	
Age (years)		82.73	[9.505]	76.78	[12.055]	<0.001
DRG ^c^ Weight		3.401	[1.464]	3.805	[6.782]	0.299
Place of residence	Rural ^d^	45 (14.71%)	(10.93–19.18)	633 (23.37%)	(21.78–25.01)	0.001
	Urban ^e^	261 (85.29%)	(80.82–89.07)	2076 (76.63%)	(74.99–78.22)	
Type of admission	Programmed	28 (9.15%)	(6.17–12.95)	183 (6.75%)	(5.84–7.77)	0.150
	Urgent	278 (90.85%)	(87.05–93.83)	2526 (93.25%)	(92.23–94.16)	
Discharge destination	Long term stay	255 (83.33%)	(78.68–87.33)	2122 (78.33%)	(76.73–79.87)	0.001
	Home	46 (15.03%)	(11.22–19.54)	366 (13.51%)	(12.25–14.86)	
	Deceased	4 (1.31%)	(0.36–3.31)	194 (7.16%)	(6.22–8.19)	
	Other ^f^	1 (0.33%)	(0.01–1.81)	27 (0.99%)	(0.66–1.45)	
Year of medical discharge	2007	42 (13.73%)	(10.07–18.09)	332 (12.25%)	(11.04–13.55)	0.939
	2008	47 (15.36%)	(11.51–19.89)	400 (14.77%)	(13.45–16.16)	
	2009	37 (12.09%)	(8.66–16.28)	336 (12.40%)	(11.18–13.70)	
	2010	35 (11.44%)	(8.09–15.55)	331 (12.22%)	(11.01–13.51)	
	2011	46 (15.03%)	(11.22–19.54)	350 (12.92%)	(11.68–14.24)	
	2012	28 (9.15%)	(6.17–12.95)	264 (9.75%)	(8.65–10.92)	
	2013	26 (8.49%)	(5.63–12.20)	252 (9.30%)	(8.23–10.46)	
	2014	23 (7.52%)	(4.82–11.06)	201 (7.42%)	(6.46–8.47)	
	2015	22 (7.19%)	(4.56–10.68)	243 (8.97%)	(7.92–10.11)	

^a^ SD, standard deviation; ^b^ 95% CI, 95% confidence interval; ^c^ DRG, diagnostic-related group; ^d^ rural: patient residing in no urban region; ^e^ urban: residents in the same region than hospital and with more than 50,000 habitants and with a density of more than 1500 residents per km^2^; ^f^ nursing homes for dependent persons.

## Data Availability

The data presented in this study are available on request from the corresponding author. The data are not publicly available due to patients’ privacy.

## Abbreviations

MBDSDH	Minimum basic data set on discharge at hospital
DRG	Diagnostic related groups
HP	Hip procedures
HCU	Hospital complexity unit
SD	Standard deviation
95% CI	95% Confidence interval

## Appendix A. Diagnosis-Related Group (DRG) Included in the Hip Procedures Group. Cantabria (Northern Spain), 2007–2015

210 (hip and femur procedures except major joints, over 17 years of age with complications) 211 (hip and femur procedures except major joint, over 17 years of age without complications) 235 (femur fractures) 236 (hip and pelvis fractures) 558 (major musculoskeletal procedures with major complication) 817 (hip replacement revision or hip replacement due to complications) 818 (hip replacement except for complications)
